# Supplementary material for: CRNNTL: Convolutional Recurrent Neural Network and Transfer Learning for QSAR Modeling in Organic Drug and Material Discovery
Source: Molecules. 2021 Nov 30;26(23):7257. doi: 10.3390/molecules26237257 (PMC8658888; doi:10.3390/molecules26237257)
Supplement: Supplementary file 1 [file molecules-26-07257-s001.zip › Supplementary Materials.pdf]

CRNNTL: convolutional recurrent neural network and transfer learning for QSAR modeling in organic drug and material discovery

The introduction of autoencoder (AE)

AE: The VAE is a developed AE. The general idea of the autoencoder is very simple and consists of setting up an encoder and a decoder as a neural network and using an iterative optimization process to learn the best encoding-decoding scheme. Thus, in each iteration, we give the autoencoder architecture (encoder and decoder) some data, we compare the encoding-decoding output with the initial data, and we update the weights of the network by back-propagating the errors through the architecture. Thus, intuitively, the whole autoencoder architecture (encoder + decoder) creates a bottleneck for the data, ensuring that only the main structural part of the message can pass and be reconstructed. From our general framework, the considered encoder family E is defined by the encoder network structure and the considered decoder family D is defined by the decoder network structure, and the encoders and decoders that minimize the reconstruction error are found by gradient descent over the parameters of these networks.

Variational autoencoder (VAE): A VAE can be defined as an autoencoder that is trained regularly to avoid over-fitting and to ensure that the latent space has good properties for the generative process. Instead of encoding the input as a single point, we encode it as a distribution over the latent space. The encoded distribution is chosen to be normal so that the encoder can be trained to return mean and covariance matrices describing these Gaussians. The reason for encoding the input as a distribution with a certain variance, rather than a single point, is that it allows a very natural representation of latent space regularization: the distribution returned by the encoder is forced to be close to the standard normal distribution. As we will see in the next subsection, we ensure local and global regularization of the latent space in this way (locally due to variance control and globally due to mean control). Thus, the loss function minimized when training the VAE is composed of a "reconstruction term" (at the last layer), which tends to make the encoding-decoding scheme as efficient as possible, and a "regularization term" (at the latent layer), which tends to regularize the organization of the latent space by making the distribution returned by the encoder close to the standard normal distribution. This regularization term is expressed as the Kulback-Leibler divergence between the returned distribution and the standard Gaussian distribution and will be further argued in the next section. We can note that the Kullback-Leibler divergence between the two Gaussian distributions has a closed form that can be directly represented by the mean and covariance matrices of the two distributions. The architecture of VAE is shown in Figure S1

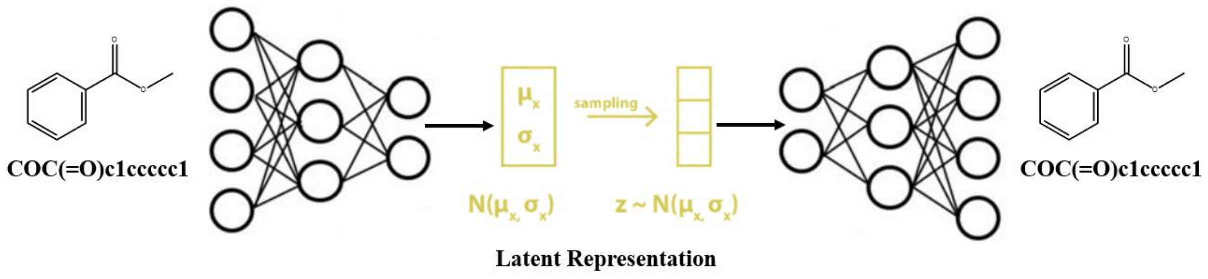

Figure S1. The architecture of the VAE

Adversarial autoencoder (AAE): AAE consists of a generative adversarial network (GAN) and a VAE. The encoder in the adversarial autoencoder is also the generative model of the GAN network. The GAN-based training ensures that the latent space conforms to some a priori latent distribution. The architecture of AAE is shown in Figure S2.

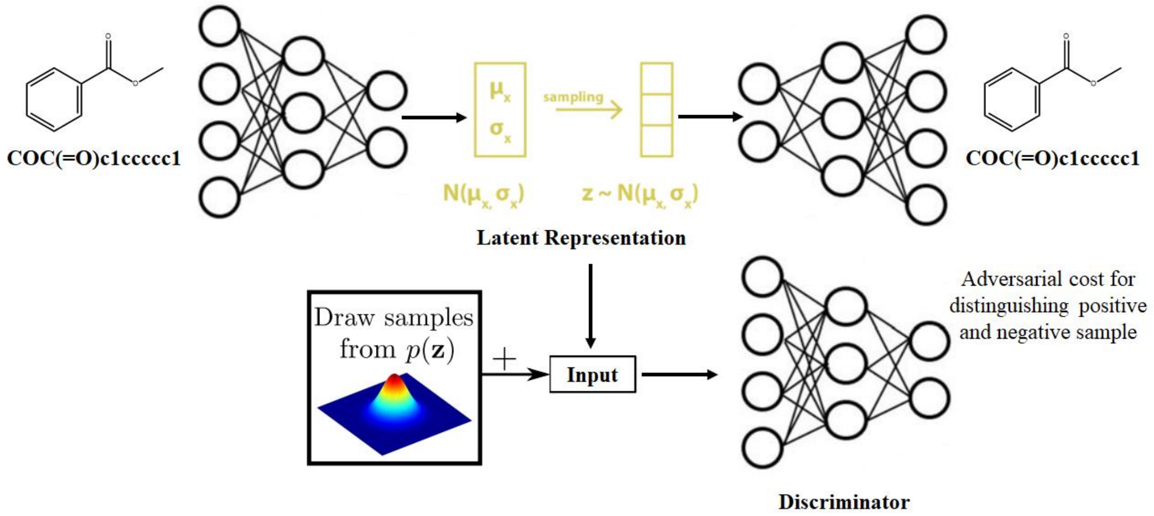

Figure S2. The architecture of the AAE

Table S1. The performance using different number of convolutional layers.

type of QSAR	3 layers	4 layers	5 layers
regression (r^2)	0.660	0.665	0.666
classification (AUC-ROC)	0.836	0.839	0.833

Table S2. The standard mean errors of CRNN and AugCRNN method for regression datasets of drug properties

Dataset	CNN	CRNN	AugCRNN	SVM
EGFR	0.01	0.01	0.01	0.01
EAR3	0.05	0.05	0.04	0.04
AUR3	0.09	0.10	0.08	0.08
FGFR1	0.02	0.02	0.02	0.03
MTOR	0.02	0.02	0.01	0.02
PI3	0.04	0.03	0.03	0.04
LogS	0.01	0.01	0.01	0.01
Lipo	0.02	0.03	0.02	0.03
BP	0.01	0.01	0.01	0.01
MP	0.11	0.10	0.08	0.10

Table S3. The standard mean errors of CRNN and AugCRNN method for classification datasets of drug properties

Dataset	CNN	CRNN	AugCRNN	SVM
HIV	0.02	0.01	0.01	0.01
AMES	0.01	0.01	0.01	0.01
BACE	0.01	0.01	0.01	0.01
HERG	0.02	0.01	0.01	0.02
BBBP	0.01	0.01	0.01	0.01
BEETOX	0.01	0.01	0.01	0.01
JAK3	0.02	0.02	0.02	0.01
BioDeg	0.02	0.03	0.02	0.02
TOX21	0.02	0.02	0.01	0.01
SIDER	0.02	0.02	0.02	0.02

Table S4. The standard mean errors of CRNN and AugCRNN method for classification datasets of material properties

Dataset	CNN	CRNN	AugCRNN	SVM
Abs_{max}	0.03	0.01	0.01	0.01
Em_{max}	0.02	0.01	0.01	0.01
Logε	0.02	0.04	0.03	0.03
σ_{abs}	0.05	0.03	0.03	0.04
lifetime	0.04	0.04	0.03	0.04

Table S5. The result of transfer learning compared with learning from scratch method.

Dataset	transfer learning	learning from scatch
MP (r^2)	0.49	0.46
SIDER (AUC-ROC)	0.73	0.70

Table S6. Transfer learning result by CNN.

Dataset	learning from scratch	FGFR1	MTOR	EGFR
PI3	0.43	0.48	0.55	0.43
AUR3	0.55	0.59	0.57	0.56

Table S7. The best result of transfer learning by freezing different parts of the neural network.

Dataset	learning from scratch	no freezing	GRU	CNN
PI3	0.47	0.61	0.48	0.61
AUR3	0.57	0.63	0.57	0.63

Table S8. The QSAR performance by the latent space derived from the AAE

Dataset	CNN	CRNN	AugCRNN	SVM
EAR3	0.23	0.35	0.38	0.25
AUR3	0.12	0.21	0.23	0.20
FGFR1	0.37	0.38	0.40	0.28
MTOR	0.37	0.39	0.35	0.41
PI3	0.08	0.17	0.18	0.16

Table S9. The QSAR performance by the latent space derived from the DDC VAE

Dataset	CNN	CRNN	AugCRNN	SVM
EAR3	0.58	0.64	0.67	0.65
AUR3	0.39	0.42	0.44	0.37
FGFR1	0.65	0.67	0.71	0.70
MTOR	0.64	0.69	0.70	0.70
PI3	0.47	0.49	0.53	0.51

Table S10. Transfer learning result by CNN.

Dataset	CDDD	AAE	DDC
PI3	30%	32%	28%
AUR3	11%	13%	10%
